# Testing the Rio Doce as a riverine barrier in shaping the Atlantic rainforest population divergence in the rodent *Akodon cursor*

**DOI:** 10.1590/s1415-47572010000400029

**Published:** 2010-12-01

**Authors:** Victor Hugo Colombi, Silvia Ramira Lopes, Valéria Fagundes

**Affiliations:** Departamento de Ciências Biológicas, Centro de Ciências Humanas e Naturais, Universidade Federal do Espírito Santo, Vitória, ESBrazil

**Keywords:** *Akodontini*, Atlantic Forest, cytochrome b, gene flow, phylogeography, Rodentia

## Abstract

*Akodon cursor* occurs in dense rainforest from northern (8° S) to southern (26° S) states along the Atlantic coast of Brazil. Previous karyological and molecular data revealed two major clades, one including northern (8-15° S) and the other southern (19-26° S) populations. The center of geographic distribution (15-20° S), which included the state of Espírito Santo, was identified as a potential vicariance region. Since river barriers are among the most discussed models in the study of Neotropical diversification, we examined whether the Rio Doce (19° S) plays an important role in shaping the population genetic divergence of *A. cursor* by including samples from Espírito Santo in the analysis. Our results showed that the northern-southern division region in Atlantic forest was no coincidence with the presence of the Rio Doce by refuting the hypothesis that this river is an effective barrier to gene flow between populations. Instead, we found evidence that isolation by geographical distance shaped the phylogeographical structure in the southern lineage. However, there is uncertainty about effectiveness of the processes involved and further studies based on wider sampling are needed.

Originally extending for 1,300,000 km^2^ along the Brazilian coast and reaching into Paraguay and Argentina, the Atlantic forest has been reduced to less than 8% of its range (Gusmão Câmara, 2003). This habitat loss has been closely linked to fragmentation but the region still harbors one of the highest percentages of endemic species in the world, with many species and even genera of vertebrates still being described (Rodrigues, 2005). Studies of the complex evolutionary history of this habitat have identified northern and southern components in the Atlantic Forest, with genetically structured populations and the differentiation of distinct groups throughout the Atlantic biome (Mustrangi and Patton, 1997; Costa *et al.*, 2000; Geise *et al.*, 2001; Pellegrino *et al.*, 2005; Cabanne *et al.*, 2007; Nogueira and Fagundes, 2008). These findings suggest that a common mechanism may have played a role in shaping the distributions of multiple taxa in this biome. Among several hypotheses for the diversification of rainforest biotas (Moritz *et al.*, 2000), evolution in palaeorefuges (Carnaval and Moritz, 2008; Thomé *et al.*, 2010) and the influence of geographical barriers (Pellegrino *et al.*, 2005; Cabanne *et al.*, 2007) are the two most commonly invoked mechanisms.

The rodent *Akodon cursor* Winge, 1887 (Cricetidae) is widely distributed in rainforest along the Atlantic coast of Brazil (Musser and Carleton, 2005), from the northern states of Paraíba and Pernambuco (8° S) through Bahia, Espírito Santo, Rio de Janeiro, Minas Gerais and São Paulo to the northern region of Paraná state in the south (26° S). This species has a peculiarly high karyotypic variability, with the diploid number varying from 2n = 14 to 16 and fundamental numbers (FN) ranging from 18 to 26 (Fagundes *et al.*, 1998). Although there is no geographic structuring of this karyological diversity, molecular studies revealed a well-structured organization of genetic diversity of mtDNA into two major clades in Atlantic forests (Nogueira and Fagundes, 2008). These authors proposed that the diversification of populations in this species was shaped by a putative geographic barrier instead of a reproductive barrier created by errors in chromosomal pairing. This suggestion was apparently supported by the fact that the 2n = 14, 2n = 15 and 2n = 16 karyotypes were equally distributed in both lineages.

By analyzing the overall geographic distribution of *A. cursor*, Nogueira and Fagundes (2008) proposed that the observed genetic variation followed the central-marginal model in which the highest genetic variability occurs in the center and declines towards the edges of the range. In their study, peripheral populations from northern and southern lineages showed the lowest intra-populational genetic diversity whereas central populations from Espírito Santo had the highest such diversity. Based on this finding, Nogueira and Fagundes (2008) suggested the existence of a contact zone between northern and southern lineages somewhere in the area of the Rio Doce basin (north of Espírito Santo), and that new samples from this region should be included in future studies.

The aim of the present work was therefore to examine the partitioning of genetic variability in *A. cursor* by including mainly samples from Espírito Santo in order to determine whether the Rio Doce is as an effective barrier for gene flow, thereby stimulating population divergence. For this, we included samples from the north and south of the Rio Doce basin that had never been tested in previous studies. Samples from São Paulo (southern lineage) and Bahia (northern lineage) were used to represent the two major lineages previously observed and to help polarize the analysis.

DNA was extracted from samples of frozen muscle or liver obtained from 64 specimens of *A. cursor* collected at 10 spawning sites in three states with Atlantic rainforest: Bahia (n = 10), Espírito Santo (n = 48) and São Paulo (n = 5) (Table 1, Figure 1), as described by Bruford *et al.* (1992). Upon extraction, a 1140 bp fragment of mitochondrial DNA containing the cytochrome-b (Cyt-b) gene was amplified using the primers described by Smith and Patton (1993). The amplifications were done in a 25 μL reaction volume containing 1X PCR buffer (Invitrogen), 1.0 mM MgCl_2_, 0.8 mM dNTPs, 0.2 mM of each primer, 3.0 U of Platinum *Taq* polymerase (Invitrogen) and 5-50 ng of template DNA. The thermal cycling conditions were: 94 °C for 3 min followed by 40 cycles of 94 °C for 30 s, 48 °C for 40 s and 72 °C for 90 s, with a final extension step of 72 °C for 5 min.

The amplicons were purified with either PureLink PCR purification kits (Invitrogen) or ExoSAP-IT® (USB Corporation), according to the manufacturers instructions, and then quantified in a NanoDrop spectrophotometer prior to cycle-sequencing. Sequencing used forward and reverse primers and was done directly in a capillary ABI3700 genetic analyser with the Big Dye Terminator protocol (Applied Biosystems). Electropherograms were inspected visually using BioEdit v. 7.09 (Hall, 1999) and alignments were done using Clustal X in MEGA v. 4 (Tamura *et al.*, 2007).

ARLEQUIN v. 3.11 (Excoffier *et al.*, 2005) was used to explore the genetic characteristics and partitioning of nucleotide diversity. The number of polymorphic sites and the haplotype (*h*) and nucleotide (π) diversity indices were computed (Nei, 1987). The appropriate model of nucleotide sequence evolution was determined using MODELTEST v. 3.7 (Posada and Crandall, 1998) and AIC parameters were chosen. Hierarchical analysis of molecular variance (AMOVA; Excoffier *et al.*, 1992) done with ARLEQUIN v. 3.11 was used to investigate the patterns of historical population structure, with the fixation indices serving to estimate the proportion of variation within populations (Φst), among the groups (Φct), and among the populations within groups (Φsc). Pairwise ΦST values were also generated for population comparisons. The amount of ongoing gene flow between populations was estimated using the Φ_ST_ values (Hudson *et al.*, 1992). To depict the hierarchical relationship between haplotypes that reveals the mutational steps involved in the transition of one haplotype to another, and to assess the association between haplotype and geography, a haplotype network was generated using the median-joining (MJ) algorithm (Bandelt *et al.*, 1999) as implemented in NETWORK 4.2.0.1 (http://www.fluxus-engineering.com/sharenet.htm). Phylogenetic analyses were done using PAUP* 4.0b1 (Swofford, 2003) with maximum-parsimony (MP). Support for individual clades was evaluated using bootstrap re-sampling (Felsenstein, 1985) with 1000 replications of random addition and tree bisection reconnection (TBR) branch swapping.

Sixty haplotypes from 64 individuals were detected across all populations (Table 1). Of 1140 sites, 205 were polymorphic, with high haplotype and low nucleotide diversity indices in all populations. There was high haplotype diversity (*h*) and low nucleotide diversity, such as is frequently encountered in populations undergoing a population bottleneck followed by demographic expansion, with the accumulation of mutations (Grant and Bowen, 1998). This feature could explain the high number of polymorphic sites and is not uncommon in Atlantic Forest species.

The MP tree topology (data not shown) and median-joining network (Figure 1) showed two major divergent lineages. The ten haplotypes of the 10 individuals from the northern lineage occurred in southern Bahia, whereas the 50 haplotypes of the 54 individuals of the southern lineage all occurred at localities in Espírito Santo (north and south of the Rio Doce) and São Paulo (Table 1), and were separated by at least 30 mutational steps. Pairwise distance differences showed no gene flow between northern (BA) and southern (ES and SP) populations, and was confirmed by the high Φ_ST_ values between BA/ES and SP populations (Table 2; Φ_ST_ > 0.63790, p < 0.05). The phylogenetic tree topology and haplotype network agreed with the clustering of the northern and southern lineages observed in the maximum parsimony analysis presented by Nogueira and Fagundes (2008).

*Akodoncursor* had a significant population genetic structure of Φ_ST_ = 0.51, and AMOVA, which was used to assess whether the Rio Doce influenced this group division (Table 2), showed that this river did not affect formation of the northern and southern lineages.

The pattern of phylogeographical groups observed in *A. cursor* could be limited by geographical landmarks, such as the Rio Doce basin, as observed in other mammals (Mustrangi and Patton, 1997; Costa *et al.*, 2000), geckos (Pellegrino *et al.*, 2005) and birds (Cabanne *et al.*, 2007), although the splitting times among clades do not suggest a common temporal origin for these patterns (Cabanne *et al.*, 2007). However, since the northern populations in Espírito Santo (Águia Branca and Governador Lindenberg) are located north of the Rio Doce, the network data supported the AMOVA results, *i.e.*, that the Rio Doce does not play an important role in the divergence of *A. cursor* lineages and therefore that this geographical barrier should not be considered as the main cause of the breakage in the dispersion of these rodents, contrary to the proposal by Nogueira and Fagundes (2008).

**Figure 1:**
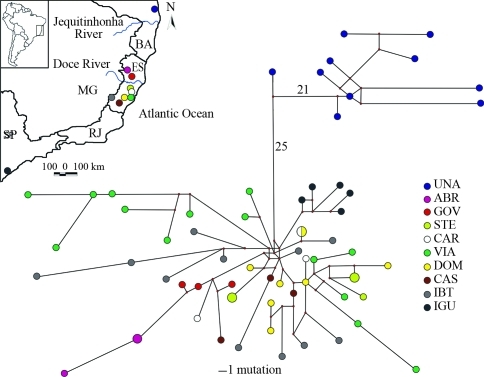
Map of localities sampled for *A. cursor* in eastern Brazil and median-joining network of haplotypes. Circle sizes are proportional to haplotype frequency and each circle is colour coded according to the haplotype's presence at the sampling locations. Unobserved haplotypes are represented by small red circles. Branches are proportional to the number of mutations, except for those with numbers, which represent highly divergent haplotypes with more than 10 mutations. Acronyms, geographic coordinates and altitude of localities are: Bahia state, municipality of Una (UNA, 15°16' S, 39°04' W, 66 m), Espírito Santo state, municipalities of Águia Branca (ABR, 18°52' S, 40°49' W, 373 m), Governador Lindenberg (GOV, 19°16' S, 40°28' W, 220 m), Santa Teresa (STE, 19°55' S, 40°36' W, 950 m), Cariacica (CAR, 20°16' S, 40°25' W, 780 m), Viana (VIA, 20°24' S, 40°29' W, 25 m), Domingos Martins (DOM, 20°22' S, 40°40' W, 701 m), Castelo (CAS, 20°36' S, 41°11' W, 262 m) and Ibitirama (IBI, 20°29'S, 41°43'W, 935-1063 m), and São Paulo state, municipality of Iguape (IGU, 24°42' S, 47°34' W, 144 m).

Additional network analysis showed that all five haplotypes from Iguape (São Paulo) grouped as a monophyletic clade (Figure 1). Despite the generally low divergence between southern (ES and SP) populations, Φ_ST_ analyses involving the Iguape (SP) and Águia Branca or Governador Lindenberg (ES) populations showed high divergence (Table 2).

Although this was no evidence of previous fragmentation in populations from São Paulo, this apparent “monophyly” may reflect either the lack of sampling of intermediate populations (including Rio de Janeiro state, for example) or the fact that the SP and ES populations are geographically far (~1000 km) from each other in the southern lineage. These observations suggest that isolation by geographical distance and the history of vicariance (geographical location) could play an important role in shaping the population genetic structure of the southern lineage, including populations from São Paulo and northern Espírito Santo.

It is well-known that sampling the extremes of a population distributed as cline can yield spurious evidence of vicariance. The data for Águia Branca and Governador Lindenberg provide further evidence of this potential isolation by distance and support the conclusion that in the geographically complex Atlantic Forest it is unlikely that forest refugia or barrier hypotheses alone will account for general patterns of lineage diversification (Thomé *et al.*, 2010). However, testing the isolation by distance mechanism requires further investigation.

Specimens with karyotypes 2n = 14 and 15 were distributed across all haplotype network branches (Table 1 and Figure 1), thus indicating that karyological data are not associated with lineage diversification, a conclusion that corroborate previous studies in *A. cursor* (Nogueira and Fagundes, 2008).

In conclusion, our data do not support a primary influence of the Rio Doce as a putative barrier in shaping the divergence between the two main mitochondrial lineages of *A. cursor* in the southeastern Atlantic forest. Rather, the data raise questions about the influence of isolation by geographical distance in modeling the phylogeographical structure of the southern lineage of *A. cursor*. However, there is uncertainty about effectiveness of the processes involved, and sampling on a wider scale is necessary to assess the importance of distinct isolation mechanisms.

## Figures and Tables

**Table 1:** Population statistics and genetic characteristics of the locations sampled for *A. cursor*. Pi – mean pairwise differences, S – number of sites with substitutions, π - nucleotide diversity, *h* - haplotype diversity and Φ_ST_ - proportion of variation within populations and SD – standard deviation. All statistics were calculated with Arlequin v. 3.11 (Excoffier *et al.*, 2005).

City, State	Acronyms	Number of individuals	Number of haplotypes	Number of polymorphic sites	Pi (SD)	S	π (SD)	*h* (SD)	Φ_ST_	Distribution of haplotypes within populations^1^	Diploid number of individuals (2n)
Una, BA	UNA	10	10	77	19.911(9.635)	79	0.0175 (0.0096)	1.000 (0.045)	0.47647	H1-H10	14, 15 and 16
Águia Branca, ES	ABR	3	2	12	8.000 (5.127)	12	0.0070 (0.0056)	1.000 (0.272)	0.55695	H54^(2)^, H55	14
Governador Lindenberg, ES	GOV	3	3	6	4.000 (2.725)	6	0.0035 (0.0029)	1.000 (0.272)	0.57400	H26-H28	14
Santa Teresa, ES	STE	5	3	16	9.000 (5.002)	17	0.0079 (0.0051)	1.000 (0.126)	0.54501	H11^(2)^, H12, H13^(2)^	14
Cariacica, ES	CAR	3	3	19	12.667 (7.922)	19	0.0111 (0.0087)	1.000 (0.272)	0.53706	H14-H16	14
Viana, ES	VIA	14	14	51	17.154 (8.122)	51	0.0150 (0.0079)	1.000 (0.027)	0.48920	H40-H53	14
Domingos Martins, ES	DOM	7	7	26	9.524 (4.980)	26	0.0083 (0.0050)	1.000 (0.076)	0.53885	H16, H20-H25	14
Castelo, ES	CAS	3	3	21	14.000 (8.718)	21	0.0123 (0.0095)	1.000 (0.272)	0.53137	H17-H19	14
Ibitirama, ES	IBI	11	11	66	19.127 (9.187)	66	0.0168 (0.0090)	1.000 (0.039)	0.47987	H29-H39	14
Iguape, SP	IGU	5	5	17	7.6000(4.275)	17	0.0067 (0.0044)	1.000 (0.126)	0.55218	H56-H60	14 and 15

^1^Number of individuals per haplotype shown in parentheses when the number of individuals > 1.

**Table 2:**
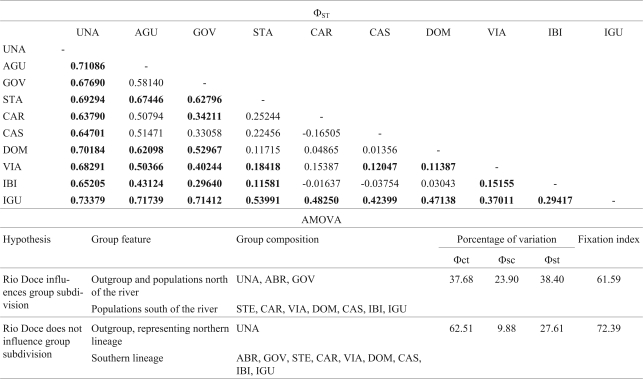
Genetic structure of the *A. cursor* populations. Upper half of table: pairwise Φ_ST_ (genetic differentiation between populations).  Lower half of table: Analysis of molecular variance (AMOVA) to test two hypothetical scenarios for the partitioning of genetic variation using the fixation indices among groups (Φct), among populations within groups (Φsc) and within populations (Φst).  Significant p-values (p < 0.05) are highlighted in bold. UNA (Bahia) was used to polarize the analysis and represent the northern lineage in *A. cursor* (see Figure 1).
